# Supplementary material for: Development of Simvastatin-Loaded Particles Using Spray Drying Method for Ex Tempore Preparation of Cartridges for 2D Printing Technology
Source: Pharmaceutics. 2023 Aug 28;15(9):2221. doi: 10.3390/pharmaceutics15092221 (PMC10537374; doi:10.3390/pharmaceutics15092221)
Supplement: Supplementary file 1 [file pharmaceutics-15-02221-s001.zip › pharmaceutics-2562134-supplementary.pdf]

pharmaceutics

Supplementary Material

Development of simvastatin-loaded particles using spray drying method for *ex tempore* preparation of cartridges for 2D printing technology

Barbara Sterle Zorec * and Rok Dreu

Faculty of Pharmacy, University of Ljubljana, Aškerčeva cesta 7, SI-1000 Ljubljana, Slovenia
* Correspondence: barbara.sterle.zorec@ffa.uni-lj.si

Abstract: In this work, a spray drying method was developed to produce drug/polymer (simvastatin/polycaprolactone) microparticles that have the potential to be used as a pre-formulation for *ex tempore* preparation of 2D printing cartridges. An experimental model was designed with the process parameters set to predict the smallest particle size, required for successful 2D printing. Three different types of particles (lactose, nanocellulose/lactose, calcium silicate) were produced, and the average size of the dry particles varied depending on the sampling location (cyclone, collection vessel). The encapsulation efficiency of simvastatin was highest with nanocellulose/lactose from the collection vessel. The one-month stability of simvastatin in the particles showed low content, but the addition of ascorbic acid as an antioxidant increased the chemical stability of the drug. Interestingly, the addition of antioxidants decreased the stability of simvastatin in the calcium silicate particles from the collection vessel. Dispersion of the particles in three different PG and water mixtures (10/90, 50/50, and 90/10% (V/V)) representing a printable ink medium with three different viscosity and surface tension properties showed that nanocellulose/lactose was the most suitable antiadhesive in terms of dispersed particle size (<1μm). After one month of storage, dispersed particles remained in the same size range without undesirable particle agglomeration.

Keywords: Inkjet printing; Spray drying; Design of experiments; Simvastatin; Antioxidants; Stability; Ink medium; Nanocellulose; Particle size; Emulsion

1. Method

1.1. *Differential scanning calorimetry*

In order to determine the physical state of SIM incorporated in particles, pure simvastatin, PCL, all three antiadhesives (lactose, NCC and CaSi), their physical mixtures and all three simvastatin-loaded particles right after preparation and after one month of storage under both storage conditions were analyzed using Differential scanning calorimetry (DSC; Mettler Toledo DSC1, Switzerland). Approximately 5 mg of sample was carefully transferred into a standard 40 μL Mettler Toledo aluminium pan, accurately weighed, covered with lid, and hermetically sealed. The closed pan was pierced just prior to the analysis to make a small pin hole. Samples were scanned in the temperature range from 5 °C to 240 °C at a heating rate of 10 °C/min. The DSC curves were normalized to the sample mass.

2. Results

2.1. *Differential scanning calorimetry*

DSC of drug-loaded particles, their respective starting materials (SIM, PCL, lactose, NCC, and CaSi), and their physical mixtures was performed (Figure S1 – S6). Indeed, simvastatin is present in its natural state in crystalline form, as a sharp melting endotherm at 138.8 °C was evident in the DSC thermogram for pure simvastatin and the corresponding physical mixtures (Figures S1, S3, and S5). However, no endothermic peak of simvastatin was observed in any of the three particle types, either immediately after spray drying or after one month of storage under either storage condition (Figures S2, S4, and S6). This confirms the transition of SIM from a crystalline to an amorphous state during spray drying. Moreover, SIM remains amorphous after one month of storage under both storage conditions.

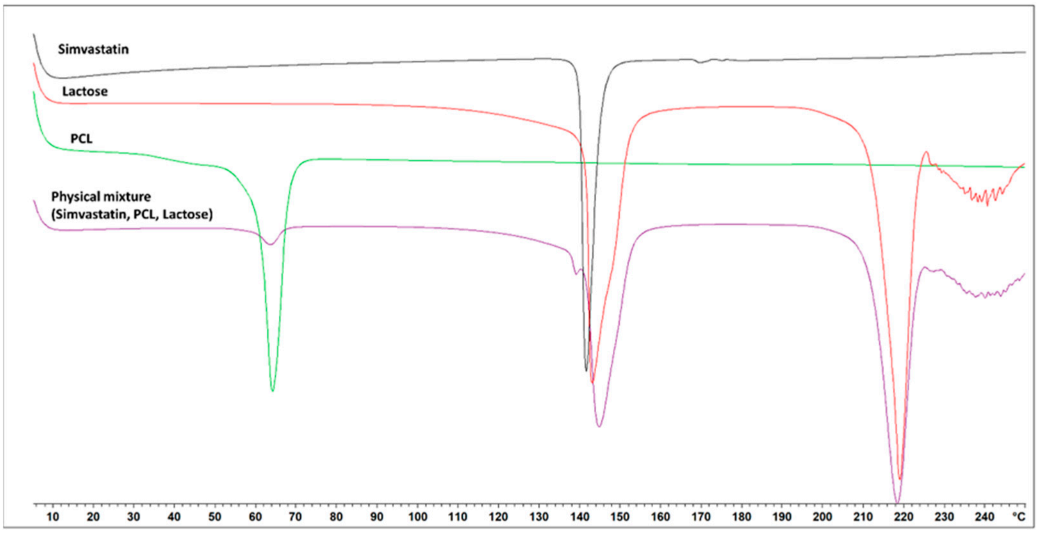

Figure S1. DSC thermogram of pure SIM, pure PCL, pure lactose, and corresponding physical mixture (of simvastatin, PCL and lactose).

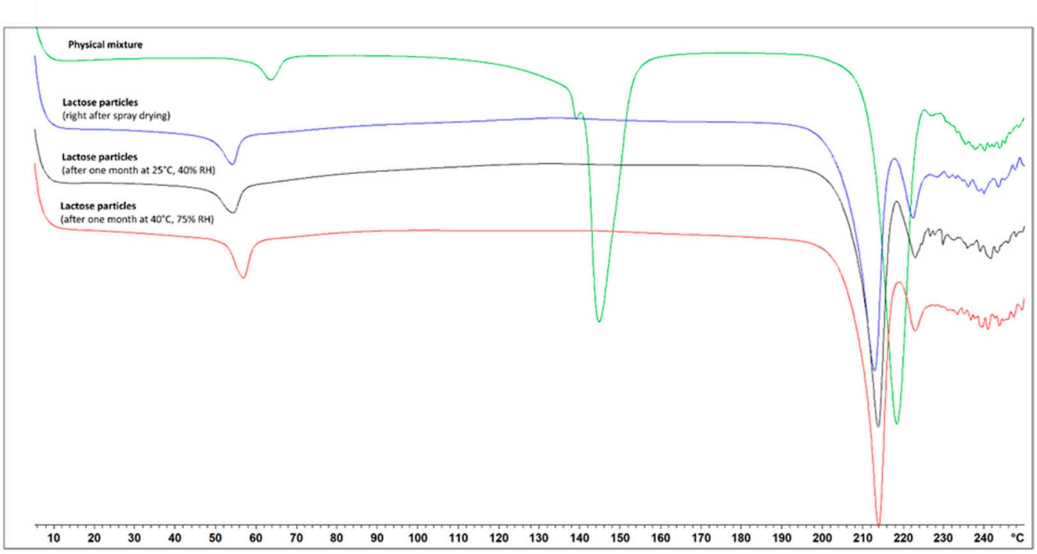

Figure S2: DSC thermogram of lactose particles immediately after spray drying, after one month of storage under both storage conditions (25 °C, 40% RH; 40 °C, 75% RH) and their corresponding physical mixture.

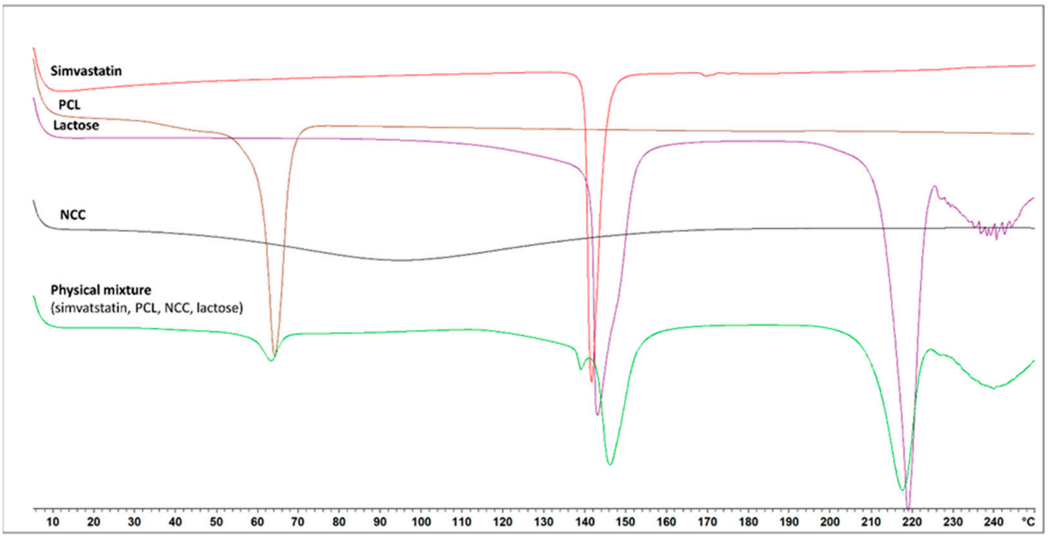

Figure S3: DSC thermogram of pure SIM, pure PCL, pure lactose, pure NCC, and their corresponding physical mixture (of simvastatin, PCL, and NCC/lactose).

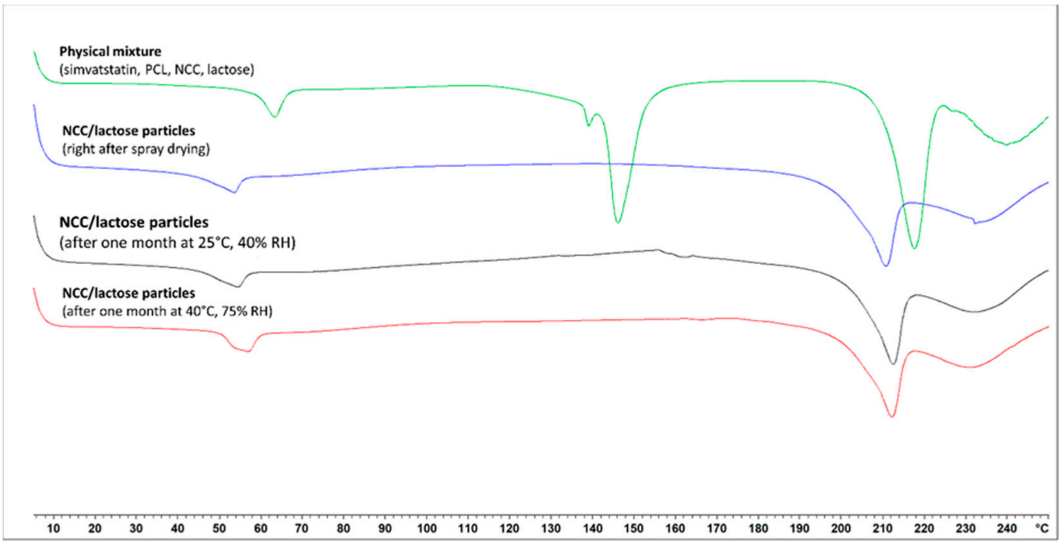

Figure S4: DSC thermogram of NCC/lactose particles immediately after spray drying, after one month of storage under both storage conditions (25 °C, 40% RH; 40 °C, 75% RH) and their corresponding physical mixture.

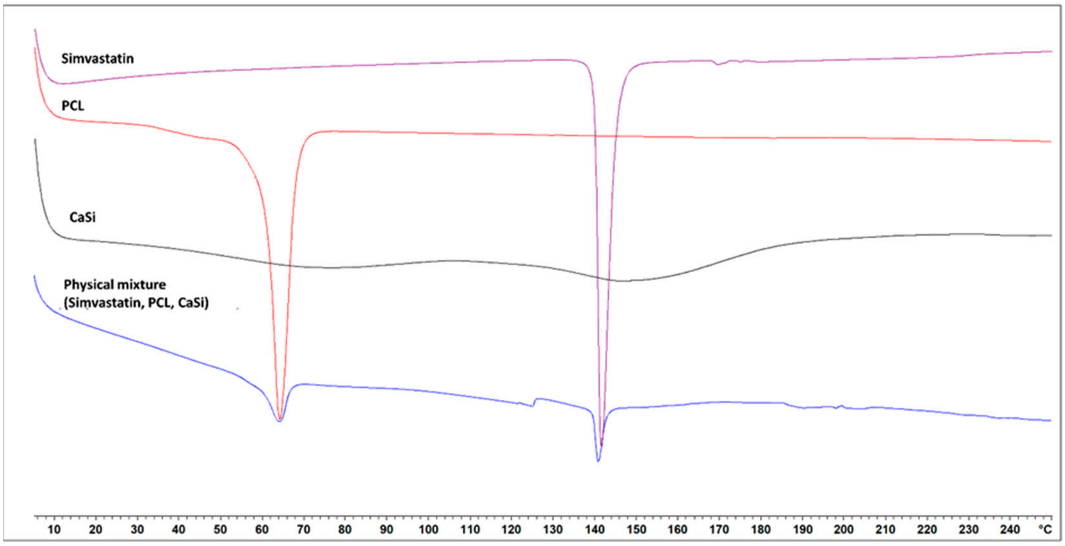

Figure S5: DSC thermogram of pure SIM, pure PCL, pure CaSi, and their corresponding physical mixture (of simvastatin, PCL, and CaSi).

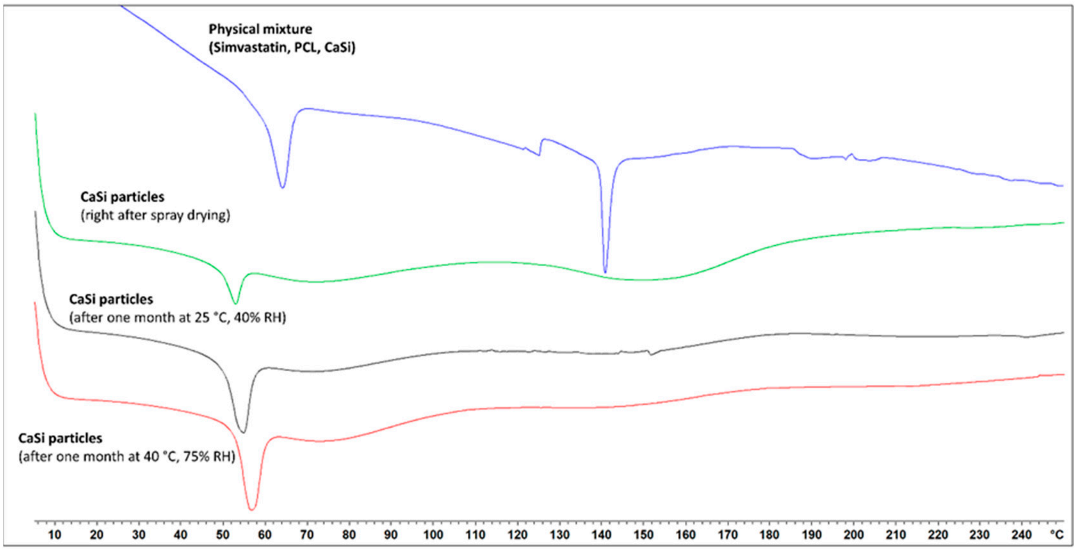

Figure S6: DSC thermogram of CaSi particles immediately after spray drying, after one month of storage under both storage conditions (25 °C, 40% RH; 40 °C, 75% RH) and their corresponding physical mixture (of simvastatin, PCL, and CaSi).
